# The Long-Term Dynamics of Shrew Communities: Is There a Downward Trend?

**DOI:** 10.3390/life14111393

**Published:** 2024-10-29

**Authors:** Linas Balčiauskas, Laima Balčiauskienė

**Affiliations:** Nature Research Centre, Akademijos 2, 08412 Vilnius, Lithuania; laima.balciauskiene@gamtc.lt

**Keywords:** insectivores, Soricidae, habitats, relative abundances, species proportions, temporal changes, Lithuania

## Abstract

Compared to other small mammals, shrews are understudied due to their limited impact on agriculture, lower biomedical importance, and difficulty to study. Based on trapping data from 1975–2023, we investigated changes in Lithuanian shrews (*Sorex araneus*, *Sorex minutus*, *Neomys fodiens*, and *Neomys milleri*) over six decades. We analyzed the relative abundance of shrews and the proportion of their species within small mammal communities to assess temporal patterns and distribution in major habitat types. The first main finding was the confirmation of a decrease in *S. araneus* abundance in the 2020s compared to the 1990s and 2010s. The species proportion in 2020s was lower than in the 1970s–2000s; the decrease started in the 1980s and accelerated in the 2000s. Abundances and proportions of *S. minutus* and *N. fodiens* showed no significant trend. The abundance of *N. fodiens* was very low. The relative abundances and proportions of *Sorex* species were highest in commensal (human-related) and mixed (including forest, wetland, and meadow) habitats. Shrews were underrepresented in agricultural habitats, with the numbers of both *S. araneus* and *S. minutus* 4.1 times lower than expected. While the presence of *S. minutus* in commensal habitats could be explained by their diet specificity, the capture of *N. fodiens* and *N. milleri* in commensal habitats is a novel feature of their ecology.

## 1. Introduction

As a mammalian group, shrews are relatively understudied compared to rodents. Possible reasons for this include the limited economic interest of this group: shrews have a limited impact on agriculture [1,2], and their biomedical importance is less than that of rodents [3,4]. Furthermore, the secretive lifestyle and high metabolic rates of several shrew species make it difficult to study them in their natural habitats [5,6,7].

Currently, the most relevant scientific publications concern the biology, ecology, and conservation of shrews. Analyses of shrew species distributions and range shifts are particularly important in the context of climate change and habitat alteration [8,9,10], especially in northern and temperate regions [11,12]. Finally, elements of interspecific competition [13,14] and effects of socioeconomic variables [15] are included in spatial models. As insectivores, shrews play various roles in natural, agricultural, and even commensal ecosystems [2]. Shrews can control invertebrate populations and participate in nutrient cycling [16,17,18,19].

The temporal variation in shrew populations has been analyzed at different temporal scales [20,21,22,23,24] or in terms of factors such as predation, food availability, and habitat quality [25,26,27].

Habitat analyses often show that shrews prefer moist environments such as wetlands, forests, and grasslands [28,29]. Studies of shrews’ habitat use and preferences indicate the importance of community composition [30], densities [31], predators, temporal factors, and landscape changes [32,33,34]. An underrepresentation of shrews in agricultural habitats has been shown in Lithuania [35]. Therefore, long-term changes in habitat preferences of shrews are important for understanding their ecology in anthropogenic landscapes [36,37]. Studies of shrews also aim to analyze the complex influence of habitat change and climate [13,38,39].

A deeper understanding of shrews’ responses to climate change or landscape dynamics requires studies of populations over longer periods, preferably decades. These studies have revealed cyclical fluctuations in various shrew species in northern latitudes [22,26] and related factors such as snow depth and soil freezing depth in winter, spring temperature conditions and winter–spring precipitation, population status, and population structure in the previous year [27]. Long-term studies on shrews represent different latitudes, from the northern ones in Siberia [21], Finland [22], and the Far East [23], to the middle [36,40,41,42,43,44] and southern latitudes [45,46]. Such studies often include the analysis of owl pellets [41,45], as they are known to be used to assess population changes in small mammals [47].

The first comparable data on shrews in Lithuania were presented in 1935–1936 [48,49]. The author listed three species of shrews: the common shrew (*Sorex araneus*), the pygmy shrew (*S. minutus*), and the water shrew (*Neomys fodiens*). However, only the localities of occurrence, the number of specimens, and their morphometric measurements were presented, without any information on the trapping effort and the habitat distribution of the shrew species.

Scientific publications on shrews started in 1975 [50], four decades later, and shrews were mainly analyzed in complex with other small mammals. The number of publications devoted exclusively to shrews is limited, and these studies analyze specific habitats, such as clearcuts [51] or orchards [35]. Two of the four shrew species identified in Lithuania, *S. araneus* and *S. minutus*, are considered more common, while *N. fodiens* is rare [52,53]. The Miller’s water shrew (*Neomys milleri*) in Lithuania was identified in 2012 [54] and was then referred to as the Mediterranean water shrew (*N. anomalus*).

The preferred habitats of *S. araneus* in Lithuania were reported to be meadows, riparian areas, reed beds, old gardens, parks, forest edges, swamps, and forests. *Sorex minutus* was reported to inhabit wetlands, meadows, and forests, while *N. fodiens* prefers water edges and adjacent habitats [52,53]. *Neomys milleri* was captured in reed and sedge habitats [54]. The reported habitat preferences were not determined on the basis of trapping analyses.

Results of the long-term dynamics of shrews with an indication of a national decline were published in 2022 [55]. The same paper reported high numbers of preyed shrews in owl pellets. However, this study did not include a habitat analysis.

The aim of the current study was to assess changes in Lithuanian shrews over six decades. We examined temporal patterns, including monthly, seasonal, and annual variations in the relative abundance of shrews and the distribution of their species within small mammal communities, as well as the relative abundance and distribution of their species among the major habitat types in Lithuania.

## 2. Materials and Methods

### 2.1. Study Site, Habitats, and Shrew Trapping

**Figure 1:**
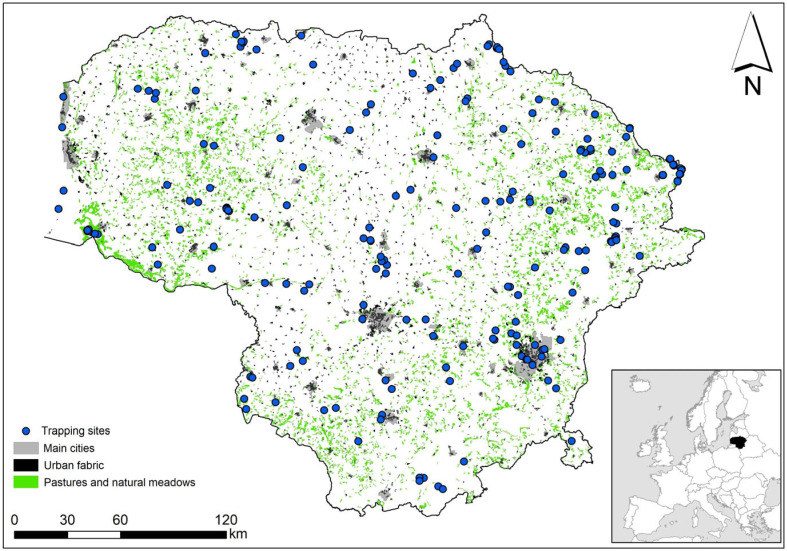
Shrew trapping sites in Lithuania, 1975–2023, with indication of meadows. CORINE land cover codes: Urban fabric: 111–112; Pastures: 231; Natural grasslands: 321 [56].

Shrew data were extracted from a small mammal trapping database covering the period between 1975 and 2023 and the entire territory of Lithuania (Figure 1). The dataset was compiled from published sources [55] and unpublished trapping protocols. Some activities, such as the inventory of mammals in protected areas (1970s–2010s), were very long-term. Some, such as the National Environmental Monitoring Program (1993–2005) and studies of flooded meadows (2004–2020), spanned more than a decade, while others lasted a decade or less. During the 1970s and 1990s–2020s, irregular and uncoordinated small mammal trapping was conducted, mostly as non-repeated events at a single site.

Small mammal trapping was conducted in a variety of habitats that we later grouped into nine categories: agricultural, commensal, disturbed, forest, meadow, mixed, riparian, shrub, and wetland. When a single trap line covered several different habitats, the combination of these fragments was characterized as a mixed habitat. At the same time, these mixed habitats are fragmented ones. A detailed description of the habitats used can be found in [57].

Shrews were trapped using the standard small mammal trapping scheme used in Lithuania—snap traps set in lines of 25 traps, 5 m apart. In most cases, the traps were set for three days and checked once a day in the morning. In some sessions, traps were set for 1–2 days or checked twice a day, morning and evening. Smaller numbers of traps were used only in commensal habitats, especially inside buildings.

Wooden 7 × 14 cm snap traps, corresponding to “The Little Nipper” by James Henry Atkinson, 1898 [58], were used in most cases. Plastic snap traps and smaller metal snap traps, as well as pedal snap traps, were used only in a few cases, so the effect of the type of trap can be considered insignificant. The standard bait was brown bread and crude sunflower oil, and only in a few trapping sessions in the 1970s–1980s was carrot, apple, or other plant material used.

**Figure 2:**
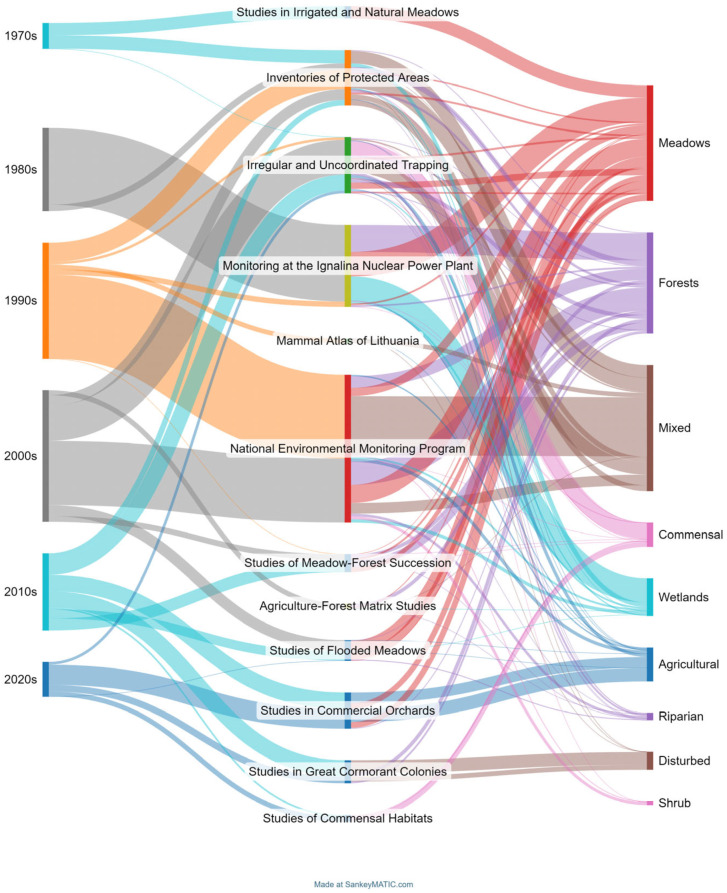
Sankey diagram [59], visualizing relations between decades, long-term projects, and habitats. Line widths depend on the trapping effort.

The relationship between decades, different short- and long-term trapping projects, and habitats is presented as a Sankey diagram [59]. In this way, we visualize flows and relationships between different categories (in our case, decades), different trapping projects (short and long term), and habitats, while at the same time providing information on the distribution of trapping effort. In the diagram shown, the thickness of the arrows indicates the strength or importance of the relationship (trapping effort) between the diagram elements. For the scale, the total trapping effort was 510,064 trap-days, trapping effort in meadows was 125,865 trap-days (Figure 2).

The presented diagram allows trends in the trapping of small mammals in Lithuania over time to be identified, indicating the most preferred trapping habitats (meadows and forests present in all decades, mixed habitats not present after 2000s, agricultural and shrub habitats in earlier decades studied with low trapping effort, etc.). Compared with the obtained results, the diagram indicates the most promising biotopes, such as commensal habitats, for future studies and allows gaps in the trapping schemes to be identified.

### 2.2. Data Processing

**Figure 3:**
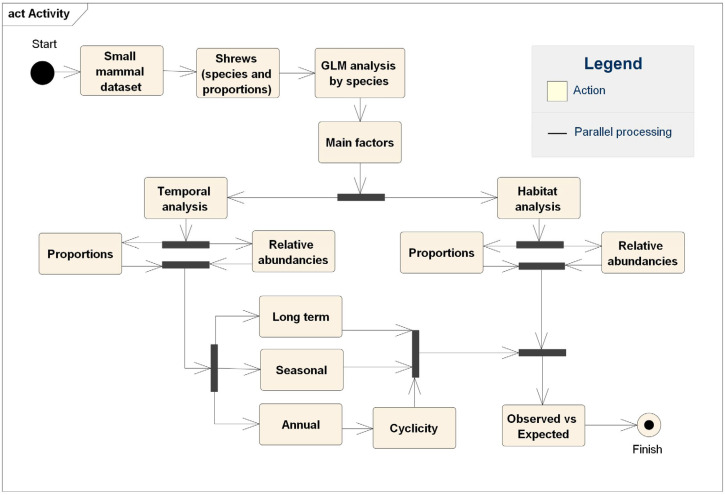
Data analysis diagram.

The sequence of data analysis is visualized in the diagram (Figure 3) and was performed separately for the three most abundant shrew species. The flow of analysis begins with the small mammal dataset, which is then narrowed to include only shrews. This narrower dataset includes data on species abundances and proportions, which are then checked for normality. Following the performance of generalized linear models (GLMs) by species and the identification of key factors, further analyses are conducted. The first of these is a temporal analysis, comprising long-term trends, seasonal variations, and annual fluctuations (including a test for cyclicity, or a recurring pattern), for both abundances and proportions. The second is a distribution analysis of abundances and proportions by habitat. The final step is the consolidation of results, in which observed values are compared with expected values derived from the temporal and habitat analyses, thus revealing any unusual patterns or outliers.

Since the dependent variables, relative abundance (RA, expressed as individuals per 1000 trap-days), and proportions in three shrew species, *S. araneus*, *S. minutus*, and *N. fodiens*, were not normally distributed, we ran six GLM models with gamma link function, as it best describes our data [60]. Year of trapping, month of trapping, and habitat were categorical factors, while trapping effort was a continuous predictor. We assessed the significance of these factors using F and *p*, and the effect size using partial eta-squared (*η*^2^). Graphs were presented as means and 95% confidence intervals (CIs). Differences in RA between categories of categorical factors were tested by post hoc analysis (Tukey HSD with unequal N). The minimum significance level was set at *p* < 0.05.

The cyclicity of shrew RA was assessed visually from the plot of annual means and confirmed by autocorrelation analysis.

PAST version 4.13 (Museum of Paleontology, Oslo College, Oslo, Norway) [61] was used for χ^2^ tests and autocorrelation, and all other calculations were performed using Statistica for Windows, version 6.0 (StatSoft, Inc., Tulsa, OK, USA) [62]. The Unified Modeling Language (UML) was used to create Figure 3 [63].

## 3. Results

### 3.1. Shrew Sample Size

Of the 57,426 small mammals analyzed, 5657 (9.9%) were *S. araneus*, 1943 (3.4%) were *S. minutus*, 230 (0.4%) were *N. fodiens*, and only three (0.005%) were *N. milleri*, formerly known as *N. anomalus*. The numbers of captured individuals and species proportions in the different decades of the study and in the different habitats are presented in Table 1. The proportions of *N. milleri* in the 2000s, 2010s, and 2020s were 0.005%, 0.009%, and 0.029%, respectively. Two of these shrews were caught in the commensal habitats, and one in the flooded meadow.

According to the number of captured individuals, *S. araneus* was generally the 5th species in Lithuania, but in the 1980s, it was the second (after the bank vole, *Clethrionomys glareolus*), and in the 1990s–2000s, it was the third (after *C. glareolus* and the yellow-necked mouse, *Apodemus flavicollis*). The other shrew species were less numerous during the whole study period [55].

### 3.2. Temporal and Habitat Factors: Results of GLM Analysis

All six GLM models were significant, but of different strength, better explaining the variation in the relative abundance and proportion of the most abundant species, *S. araneus*. The overall model statistics are presented in Table 2.

Two temporal factors (year and month of study) and the habitat where shrews were trapped had different effects in the models of all three shrew species, influencing the variation in both RAs and species proportions (Table 3). Trapping effort was not significant in all models except the RA of *S. araneus* and, even in this model, it was too weak compared to other factors.

Based on the GLM results, we further analyzed the temporal dynamics of shrew RA and proportions, and the habitat dependence of these indices by species.

### 3.3. Temporal Dynamics of Shrew Species

Throughout the study period and independent of habitat, the RA of *S. araneus* was 7.8 ± 0.4 (range: 0–180.0) ind./1000 trap-days, that of *S. minutus* 2.5 ± 0.2 (0–121.2) ind./1000 trap-days, and that of *N. fodiens* 0.3 ± 0.04 (0–50.0) ind./1000 trap-days. Correspondingly, the average species proportions in the small mammal communities were 7.54 ± 0.38% (0–100%) for *S. araneus*, 1.92 ± 0.17% (0–100%) for *S. minutus*, and 0.15 ± 0.03% (0–50%) for *N. fodiens*.

**Figure 4:**
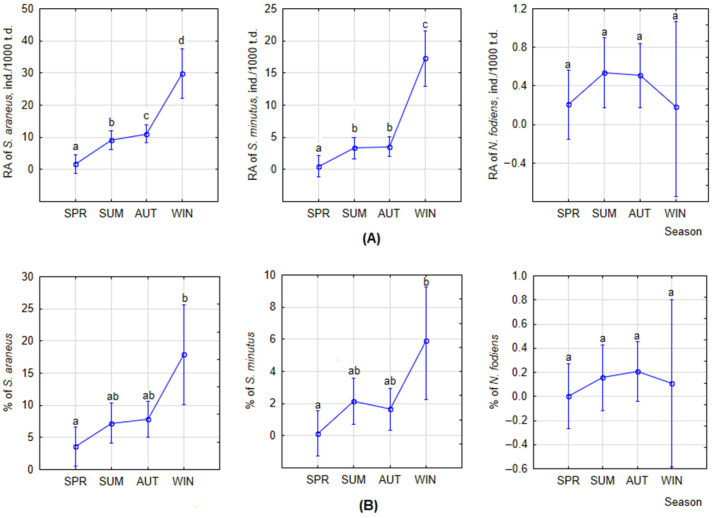
Changes in RAs (**A**) and proportions (**B**) of three shrew species by season, calculated for covariates at their means; t.d.—trap-days. Statistically significant differences are indicated by different letters.

The monthly dynamics of shrew RA show pronounced variations. In *S. araneus*, RA was high in December (25.9 ± 13.3 ind./1000 trap-days), January (41.8 ± 15.1), and February (29.6 ± 12.3), with the second peak in August (28.2 ± 4.4 ind./1000 trap-days). In *S. minutus*, the highest RA was also in winter months, December and January (22.9 ± 12.2 and 22.2 ± 8.0 ind./1000 trap-days, respectively). The second peak in August and September in this species was much lower (5. ± 1.2 and 6.0 ± 1.0 ind./1000 trap-days, respectively). The highest RA in *N. fodiens* was recorded in August and January, 1.7 ± 0.7 and 1.4 ind./1000 trap-days, respectively. Summarized by season, the highest RA of *S. araneus* and *S. minutus* was in winter (Figure 4A), significantly exceeding those in other seasons (post hoc, *p* < 0.0001). The differences between RA in spring, summer and fall were all significant in *S. araneus* (post hoc, *p* < 0.05) and all except that between summer and fall in *S. minutus* (post hoc, *p* < 0.05). In *N. fodiens*, the RA between seasons was not different (Figure 4A).

The seasonal dynamics of shrew species proportions in small mammal communities followed those of RA (Figure 4B), but the only significant difference was between the winter and spring proportions of *S. araneus* and *S. minutus* (Figure 4B).

**Figure 5:**
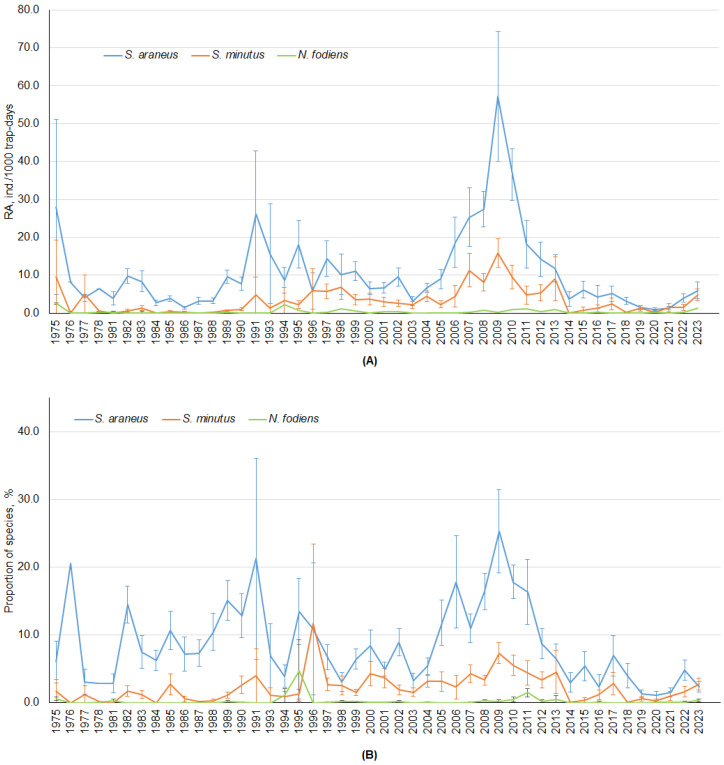
Annual changes in RAs (**A**) and proportions (**B**) in three shrew species, calculated for covariates at their means.

The annual shrew RA did not show cyclic short-term fluctuations (Figure 5A), although, in *S. araneus*, we visually observed peaks in abundance at about 20 year periods. In *S. minutus*, these long-term changes were also present, but less pronounced. Similarly, the proportion of these species in the community followed the same pattern of irregular fluctuations and a peak representation in periods shorter than 20 years (Figure 5B). While year-to-year differences were not significant, the minimum and maximum RA as well as proportions differed (post hoc, *p* < 0.001).

**Figure A1:**
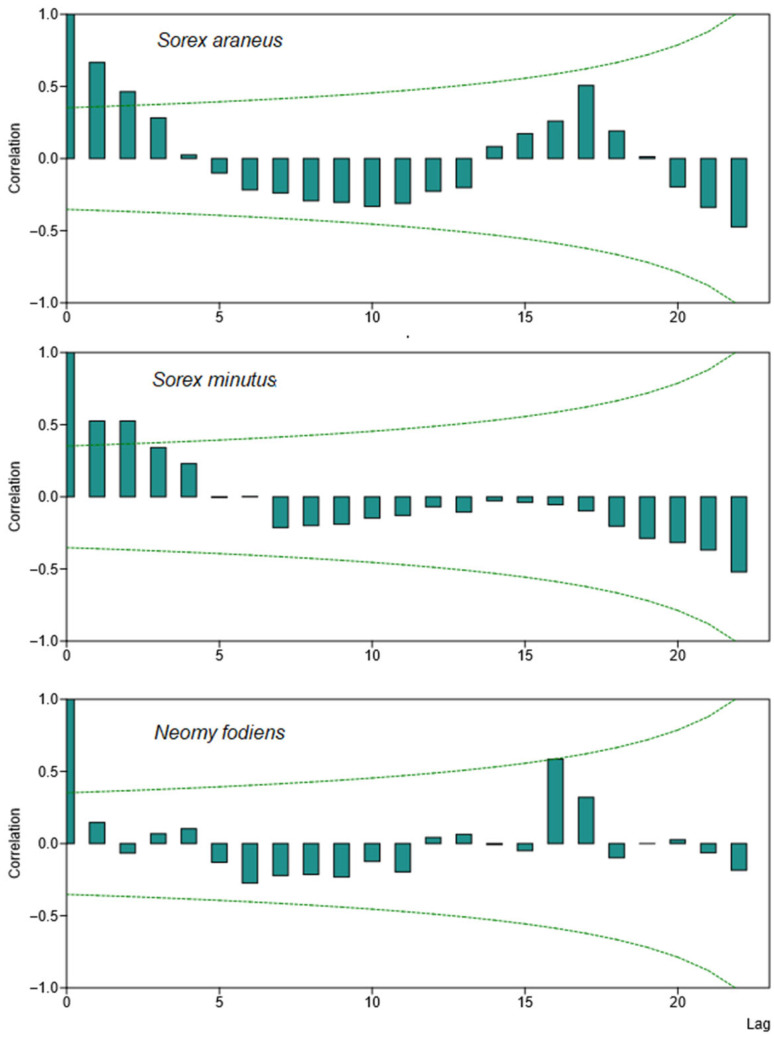
Autocorrelation of annual relative abundances of *Sorex araneus*, *Sorex minutus*, and *Neomys fodiens* with different lag periods. Lines show 95% confidence limits.

The absence of regular RA fluctuations in shrews was confirmed by autocorrelation analysis (Figure A1). In *S. araneus* and *S. minutus*, there were a few one- to two-year oscillations, but the range of RA differences was small (Figure 5A).

**Figure 6:**
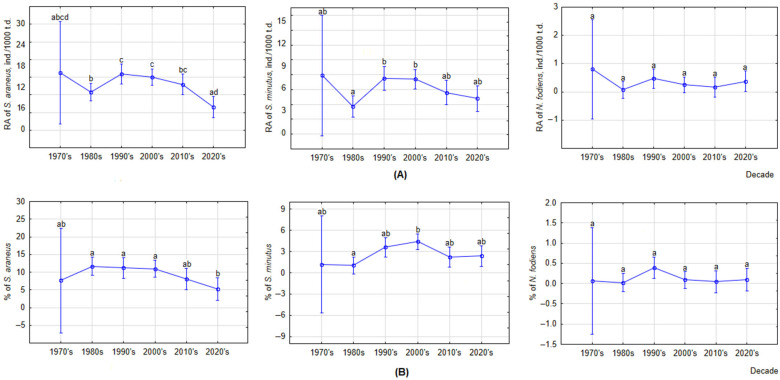
Changes in RAs (**A**) and proportions (**B**) of three shrew species by decade, calculated for covariates at their means; t.d.—trap-days. Statistically significant differences are indicated by different letters.

Analyzed by decade and including covariates at their means, the RA of *S. araneus* decreased in the 2020s, being significantly lower than in the 1990s–2010s (post hoc, *p* < 0.01). Compared to the 1990s, the RA decreased 4.0 times, compared to the 2000s—3.5 times, and compared to the 2010s—3.2 times. The minimum RA was observed in the 1980s, followed by a significant increase in the 1990s–2000s (*p* < 0.0001). In the period of the 1990s–2010s, the RA of *S. araneus* was stable (Figure 6A). In *S. minutus*, the increase in RA in the 1990s–2000s was significant compared to the minimum in the 1980s (post hoc, *p* < 0.0001), and the following decrease in the 2010s–2020s was not significant. The RA of *N. fodiens* did not differ by decade and was very low.

The proportion of *S. araneus* was significantly lower in the 2020s than in the 1970s–2000s (post hoc, *p* < 0.01), with the decline starting in the 1980s and accelerating in the 2000s (Figure 6B). The proportion of *S. araneus* in the 2020s, compared to the 1970s, decreased by 4.4 times, in the 1980s by 4.2, in the 1990s by 4.0, in the 2000s by 3.4, and in the 2010s by 2.3 times. The proportion of *S. minutus* was stable, with the only significant difference found between the 1980s and 2000s (post hoc, *p* < 0.0001). The proportion of *N. fodiens* did not differ by decade and was very low.

**Figure 7:**
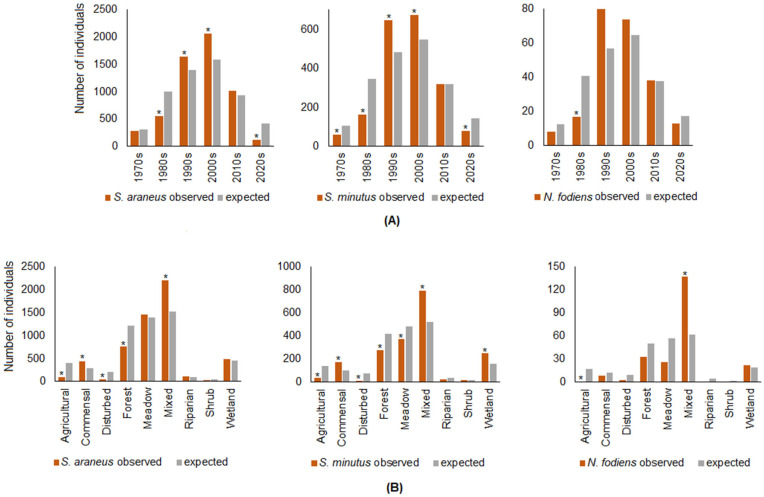
Observed and expected numbers of shrews by decade (**A**) and habitat (**B**). Statistically significant differences between numbers are indicated by asterisks.

The observed (trapped) and expected numbers of shrews by decade differed significantly for *S. araneus* (χ^2^ = 616.5, df = 5), *S. minutus* (χ^2^ = 228.4), and *N. fodiens* (χ^2^ = 27.4), all at *p* < 0.001. The underrepresentation of *S. araneus* was most pronounced in the 1980s (when the trapped numbers were 1.8 times less than expected) and 2020s (3.8 times less), while in the other decades, the numbers were over- or underrepresented 1.1–1.3 times (Figure 7A). In *S. minutus*, the captured numbers were less than expected in the 1970s (1.7 times less), 1980s (2.1 times less), and 2020s (1.8 times less). In the 2010s, the expected and observed numbers were the same, while in the 1990s and 2000s, we trapped 1.2–1.3 times more *S. minutus* than expected based on trapping effort (Figure 7A). The underrepresentation of *N. fodiens* occurred in the 1970s and 1980s (1.6–2.4 times each) and in the 2020s (1.3 times), while in the 1990s, the species was overrepresented 1.4 times, and in the 2000s and 2010s, the numbers of *N. fodiens* were as expected (Figure 7A).

### 3.4. Relative Abundance and Proportion of Shrews in Different Habitats

**Figure 8:**
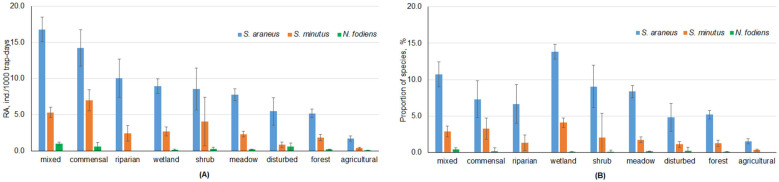
Relative abundances (**A**) and proportions (**B**) of shrew species in the investigated habitats.

The highest RAs of *S. araneus* were observed in mixed and commensal habitats (16.8 ± 1.7 and 14.2 ± 2.5 ind./1000 trap-days, respectively), while the lowest RAs were found in agricultural and forest habitats (1.7 ± 0.4 and 5.2 ± 0.6 ind./1000 trap-days, respectively). The differences between these groups were all significant (post hoc, *p* < 0.0001). The highest RAs of *S. minutus* were observed in commensal and mixed habitats (7.0 ± 0.5 and 5.3 ± 0.7 ind./1000 trap-days, respectively), and the lowest in agricultural and disturbed habitats (0.4 ± 0.1 and 0.8 ± 0.4 ind./1000 trap-days, respectively); the differences between the groups were significant at *p* < 0.05. The highest RA of *N. fodiens*, 1.0 ± 0.2 ind./1000 trap-days, was observed in mixed habitats, while the species was not found in riparian habitats (Figure 8A).

The observed and expected numbers of shrews captured by habitat differed significantly for *S. araneus* (χ^2^ = 912.7, df = 8), *S. minutus* (χ^2^ = 458.5), and *N. fodiens* (χ^2^ = 140.6), all *p* < 0.0001.

In mixed and commensal habitats, the number of captured *S. araneus* was 1.5 times higher than expected, while in agricultural habitats, it was 4.1 times, in disturbed habitats, 3.9 times, and in forests, 1.6 times lower than expected. In the other habitats, the representation of shrews was similar to that expected from the trapping effort, with differences of 1.1–1.3 times (Figure 7B). In mixed habitats, wetlands, and commensal habitats, the number of *S. minutus* caught was 1.5, 1.6, and 1.7 times higher than expected. This species was mostly underrepresented in disturbed and agricultural habitats (6.2 and 4.1 times lower than expected), and also in forest and riparian habitats (1.5 times lower). *Neomys fodiens* was only overrepresented in mixed habitats, while in wetlands, the observed numbers were close to those expected. In other habitats, *N. fodiens* was underrepresented, especially in disturbed (4.4 times less) and agricultural (16.6 times less) habitats (Figure 7B).

The highest proportions of *S. araneus* in small mammal communities were observed in wetlands and mixed habitats at 13.9% and 11.1%, respectively. In agricultural areas, the proportion of *S. araneus* was only 1.5%, significantly less than that observed in wetlands, mixed habitats, and meadows (post hoc, *p* < 0.005). In *S. minutus*, the highest proportion in the small mammal community was found in wetlands and commensal habitats at 4.1% and 3.5%, respectively, significantly exceeding the minimum in agricultural habitats at 0.4% (post hoc, *p* < 0.05). In *N. fodiens*, the species proportions in the small mammal communities were similar, with a maximum of 0.4% in mixed habitats (Figure 8B).

## 4. Discussion

The study of the temporal dynamics of shrew species and their habitat preferences based on long-term trapping data revealed two main findings: (1) a decrease in the abundance of *S. araneus* in the last decade, and (2) habitat preferences that differ from those reported in previous publications.

We found that monthly indices of relative abundance, being highest in the period from December to February in *S. araneus* and from December to January in *S. minutus*, ensured that, for both species, the RA was significantly higher in winter than in other seasons. In *N. fodiens*, there was no seasonal effect in terms of relative abundance.

At high latitudes (62° N), Siberian *S. araneus* and *S. minutus* showed several peaks in RA during the year, with the main one between August and September, and earlier peaks between June and July and between July and August [21]. The decline in shrew numbers was related to frosts with low snow cover (especially in early winter) causing mass mortality [37]. In Finland (62.5–69° N), the decline in shrew numbers was clearly expressed in summer and autumn [22].

In Central Europe (mid-latitude, 49–50° N), the maximum numbers of *Sorex* shrews were observed in October [44], but trapping did not continue after November. In southern England (51° N), the maximum numbers of *S. araneus* and *S. minutus* were observed in summer, declining into winter when all adults died [64], but earlier work by this author indicated an underestimation of shrews in the winter period [65]. Further south, in Austria (47.8° N), the maximum abundance of *S. araneus* was observed in November [41].

Therefore, the seasonal dynamics of shrew numbers is certainly related to latitude and, as shown by the authors quoted above [21,22,41,44,64,65], depends on various climatic factors. The advantage of our study was that, unlike these authors, it also analyzed material from winter trappings. The same latitudinal effect is related to the cyclicity of shrew numbers: reported regular 4-year cycles of different shrew species in Siberian high latitudes [21] were partially confirmed by [27], but not in mid-latitudes [44,64]. However, 3-year-long cycles of *S. araneus* were found in the specific habitat (strips of open habitat within a temperate forest) in Poland, 52.7° N [40]. We also did not find regular shrew cycles (see Figure 5A).

We found a significant decrease in the RA and proportion of *S. araneus* in the 2020s, while RA and the proportion of *S. minutus* was stable after the 2000s. No long-term changes in RA and proportions were observed in *N. fodiens*. However, although the trapping effort in the 2020s was 37,860 trap-days, this decade is only represented by three years in our study, so the observed decline in *S. araneus* abundance over this period should be confirmed by further studies. In line with [14], analyses should include climatic dynamics and all shrew species to take into account the simultaneous influence of weather and interspecific competition.

Long-term studies of owl diets show that the proportion of shrews varies, as does their representation in the owl diet in different time periods. In Austria, the abundance of *S. araneus* and *S. minutus* in the period 2004–2016 showed irregular fluctuations, but no decrease was found in the diet of barn owls (*Tyto alba*) [41]. In Slovenia (46° N), the shrew proportions remained similar during 1982–2001, and the author assumed that *T. alba* avoided shrews as a food type [66]. However, this was not confirmed by W.R. Meek et al. in the UK [47], where *S. araneus* was the third most important prey item for *T. alba*, accounting for 20.5% of the diet. In Italy (45° N), a strong decrease in the frequency of occurrence of *S. araneus* (2.44 times) and the bicolored white-toothed shrew, *Crocidura leucodon* (3.14 times), in the prey of different owl species was reported between the periods 1994–1995 and 2015–2016 [46]. The decrease in shrews in the diet of *T. alba* over 40 years (1972–2012) in Italy was confirmed by [45]. An analysis of owl pellets in Lithuania did not confirm the significance of the decrease in *S. araneus* in the tawny owl (*Strix aluco*) diet between the periods 1991–2000 and 2001–2010 [55]. Therefore, no clear conclusion can be drawn for the whole of Europe from the changes in the number of shrews in the owl diet.

We found the highest RA of *S. araneus* and *S. minutus* in mixed or commensal habitats, while significantly lower RAs of both species were found in agricultural habitats (see Figure 8A). The underrepresentation of both species in agricultural habitats was very strong, and the number of shrews captured was 4.1 times lower than expected. As for *N. fodiens*, only mixed habitats were characterized as favorable. The latter phenomenon was explained as a sustainable coexistence of *N. fodiens* with *S. araneus* in dry habitats and near water, maintained by fluctuations in species abundance [67]. The importance of mixed habitats and ecotones for *S. araneus* and *S. minutus* was confirmed in Moldova for the period 2003–2016 [68].

The same four shrew species present in Lithuania were studied for coexistence along a small stream in the Białowieża Forest in eastern Poland, at a similar latitude. It was found that the separation of habitat niches was based less on macrohabitat and plant cover than on distance to water and ground wetness [29]. There are simply no other studies that can explain the patterns of habitat use we found in shrews. Other authors either examined detailed aspects of the use of a single habitat or the details of interspecific habitat sharing.

In low latitudes, e.g., in the Apennines of Italy, five shrew species were found to coexist in a fragmented landscape through habitat and temporal partitioning between species of similar size, thus reducing competitive pressure [69]. While the authors suggest including measurements of the vegetation structure of habitat patches when studying the distribution of shrews in fragmented landscapes, this is not possible in our case as the study is based on retrospective data. Spatial and temporal niche segregation has been confirmed as a factor of coexistence of shrews at low latitudes in the Gulf of Cádiz [70]. The other factors of coexistence in small mammal assemblages in low latitudes have been shown to be soil parameters [2] and changes in land use [71].

In our study, the highest proportion of *S. araneus* was observed in small mammal communities from wetlands and mixed habitats, and that of *S. minutus* in wetlands and commensal habitats. The lowest proportions of both *Sorex* species were found in agricultural habitats. The ratio between the abundance of these species in all listed habitats independent of biotope was 2.91:1. Significantly more *S. minutus* were caught in Moldova, where the ratio between *S. araneus* and *S. minutus* in the period 2003–2016 was 1.37:1 [68]. In Poland, the dominance of *S. araneus* over *S. minutus* was observed in three biotopes between 1977 and 2006, but the authors did not quantify it [36]. The other Polish studies show that the ratio between *S. araneus* and *S. minutus* is 4.6:1 in three productive habitats, tussock-sedge swamp, streamside alder–ash forest, and the ecotone between them [29], and about 4.7:1 in meadows and river valleys [40].

In the productive habitats of Finland and Karelia, the ratio between *S. araneus* and *S. minutus* ranged from 2.42:1 in pine forest to 6.5:1 in meadow and 8.7:1 in bog ecotones. In contrast, unproductive barren forest habitats were characterized by a ratio where *S. minutus* was equal or even dominant over *S. araneus*, ranging from 0.69:1 to 1.09:1 [72]. We therefore assume that the ratio between *S. araneus* and *S. minutus* could be used as a measure of habitat productivity for Soricidae, at least in mid-latitudes. However, as shown in [21], this ratio may fluctuate seasonally and in the long term, and, thus, may be erroneous in short-term observations.

In the boreal forests of middle and high latitudes in Russia, it was shown that the shrew community changed from a dominance of small species in unproductive habitats to a dominance of larger shrew species in productive habitats. The structure of the shrew community is then considered to be a proxy for habitat productivity [73]. Similar observations of the larger and more competitive shrew species being most abundant in the more productive habitats have been published previously for Finland, with the hypothesis of increasing productivity of forest habitats from north to south [72]. In Lithuania, however, the smaller *S. minutus* was most abundant in wetlands, commensal habitats, and mixed habitats (Figure 8B). As reported from the Middle Urals, the main characteristic of habitat quality in coniferous forests is the abundance of invertebrates [74]. We have no data to test whether this is also true for Lithuania.

The complexity of shrew surveys, differences in species composition, and the ecological characteristics of individual species at different latitudes lead to the lack of a uniform response of these animals to climate change, habitat characteristics, and anthropogenic impacts. For example, the response of *S. araneus* to deforestation is generally more positive than that of *S. minutus* [33]. *Sorex araneus* prefers moist forests and deep soils [75]. Both species respond positively to long-term habitat protection [42]. Despite being one of the most common small forest mammal species [29,53,65,73,75], the very low abundance of *S. araneus* in certain forest habitats for almost a decade could raise the question of species preferences [76].

While some authors indicate a sensitivity of *S. araneus* to high grazing intensity on meadows [32], we found a decreasing trend of this species at medium grazing and mowing intensity [43]. On the other hand, *S. minutus* has been reported to use anthropogenic habitats (which we call commensal habitats) as a food source [77]. Our data show that the relative abundances of not only *S. minutus* but also *S. araneus* are highest in commensal habitats. Despite very low abundances, two species of water shrew, *N. fodiens* and *N. milleri*, were found for the first time in Lithuania in homesteads, including outbuildings [35]. Studies on shrews in commensal habitats are very limited and need further attention.

We agree with the position expressed in [14] that climate is not the only factor explaining differences in shrew distribution and ecology. Studies should include all shrew species present in the locality/region, and results must be related to climatic gradients or latitudes. It is possible that climatic effects have a stronger influence on shrews at higher latitudes [78]. The evaluation of shrew communities not only in forests, but also in other habitats simultaneously, along with studies of their diets, is highly desirable to understand recent changes in the habitat distribution of shrew species in response to anthropogenic transformations.

Currently, none of the four species of shrews are protected in Lithuania as they are not threatened and do not require special management measures. In the future, as climate change and habitat anthropogenicity act together, the situation may change, and the current status of the species shown in this publication can therefore be used as a starting point for assessing changes.

## 5. Conclusions

The long-term temporal dynamics of shrews in Lithuania were species-specific. While the RA of *S. araneus* significantly decreased in the 2020s compared to the 1990s–2010s, in *S. minutus*, the RA decrease in the 2010s–2020s was not significant, and the RA of *N. fodiens* by decade was stable and very low.

The proportion of *S. araneus* in small mammal communities in the 2020s was significantly lower than in the 1970s–2000s. Its decline started in the 1980s and accelerated in the 2000s. The proportions of *S. minutus* by decade were stable, while those of *N. fodiens* were stable and very low.

The habitat preferences of shrews in Lithuania are not typical. The highest RAs of *S. araneus* were observed in mixed and commensal habitats, and the lowest in agricultural and forest habitats. The highest RAs of *S. minutus* were observed in commensal and mixed habitats, and the lowest in agricultural and disturbed habitats. The highest RA of *N. fodiens* was observed in mixed habitats. The numbers of both *S. araneus* and *S. minutus* in agricultural habitats were 4.1 times lower than expected. This habitat is characterized by a low proportion of both listed species in small mammal communities.

Records of *N. fodiens* and *N. milleri* in commensal habitats of Lithuania are a new feature of their ecology.

## Figures and Tables

**Table 1:** Distribution of shrew sample sizes across decades, seasons and habitats. *N*—number of trapped individuals, %—proportion of the species among all trapped small mammals.

Decade/Habitat	*Sorex araneus*		*Sorex minutus*		*Neomys fodiens*	
	*N*	%	*N*	%	*N*	%
1970s	285	7.8	61	1.7	8	0.2
1980s	555	12.4	164	3.7	17	0.4
1990s	1640	10.0	645	3.9	80	0.5
2000s	2057	11.2	674	3.7	74	0.4
2010s	1009	9.2	319	2.9	38	0.3
2020s	111	3.2	80	2.3	13	0.4
Agricultural	99	3.9	34	1.3	1	0.04
Commensal	436	8.7	174	3.5	8	0.2
Disturbed	55	2.7	12	0.6	2	0.1
Forest	768	6.6	273	2.3	33	0.3
Meadow	1454	12.7	370	3.2	26	0.2
Mixed	2209	10.9	794	3.9	137	0.7
Riparian	106	8.8	23	1.9		
Shrub	36	10.2	17	4.8	1	0.3
Wetland	494	16.9	246	8.4	22	0.8

**Table 2:** Statistics of GLM models with year of trapping, month of trapping, and habitat as categorical factors, and trapping effort as continuous predictor on relative abundance (RA) of shrews and their proportion (%) in small mammal communities. Degrees of freedom (df = 921,474) were the same for all models.

Species	Index	F	*p*	R^2^
*Sorex* *araneus*	RA	7.88	<0.0001	0.288
	%	2.23	<0.0001	0.067
*Sorex* *minutus*	RA	4.65	<0.0001	0.176
	%	2.05	<0.0001	0.058
*Neomys* *fodiens*	RA	1.50	<0.01	0.028
	%	1.83	<0.0001	0.047

**Table 3:** Effects of trapping effort, decade, season, and habitat on relative abundance (RA) of shrews and their proportion (%) in small mammal communities. Significance levels: ***—*p* < 0.001; **—*p* < 0.01; *—*p* < 0.05; ^NS^—non significant.

Species	Index	Trapping Effort	Year	Month	Habitat
*Sorex* *araneus*	RA	F = 10.3 ***, *η*^2^ = 0.007	F = 5.0 ***, *η*^2^ = 0.166	F = 9.8 ***, *η*^2^ = 0.138	F = 5.5 ***, *η*^2^ = 0.029
	%	F = 0.0 ^NS^, *η*^2^ = 0.000	F = 1.5 **, *η*^2^ = 0.056	F = 1.6 *, *η*^2^ = 0.024	F = 5.5 ***, *η*^2^ = 0.030
*Sorex* *minutus*	RA	F = 2.4 ^NS^, *η*^2^ = 0.002	F = 3.3 ***, *η*^2^ = 0.116	F = 4.8 ***, *η*^2^ = 0.072	F = 2.6 **, *η*^2^ = 0.014
	%	F = 0.5 ^NS^, *η*^2^ = 0.000	F = 1.4 *, *η*^2^ = 0.055	F = 1.5 *, *η*^2^ = 0.024	F = 4.8 ***, *η*^2^ = 0.025
*Neomys* *fodiens*	RA	F = 0.1 ^NS^, *η*^2^ = 0.000	F = 0.9 ^NS^, *η*^2^ = 0.033	F = 2.0 **, *η*^2^ = 0.032	F = 0.9 ^NS^, *η*^2^ = 0.004
	%	F = 0.7 ^NS^, *η*^2^ = 0.000	F = 2.3 ***, *η*^2^ = 0.085	F = 0.8 ^NS^, *η*^2^ = 0.014	F = 0.6 ^NS^, *η*^2^ = 0.003

## Data Availability

This is ongoing research; therefore, data are available from the corresponding author upon request.
